# Protective effect of sterubin against neurochemical and behavioral impairments in rotenone-induced Parkinson's disease

**DOI:** 10.1590/1414-431X2023e12829

**Published:** 2024-02-09

**Authors:** M.M. Alqurashi, F.A. Al-Abbasi, M. Afzal, A.M. Alghamdi, M. Zeyadi, R.A. Sheikh, S. Alshehri, S.S. Imam, N. Sayyed, I. Kazmi

**Affiliations:** 1 Department of Biochemistry, Faculty of Sciences, King Abdulaziz University, Jeddah, Saudi Arabia; 2 Department of Pharmaceutical Sciences, Pharmacy Program, Batterjee Medical College, Jeddah, Saudi Arabia; 3 Experimental Biochemistry Unit, King Fahd Medical Research Center, King Abdulaziz University, Jeddah, Saudi Arabia; 4 Department of Pharmaceutics, College of Pharmacy, King Saud University, Riyadh, Saudi Arabia; 5 School of Pharmacy, Glocal University, Saharanpur, India

**Keywords:** Flavonoids, Neuroprotective, Neuroinflammation, Progressive nerve damage

## Abstract

This study was conducted to evaluate how sterubin affects rotenone-induced Parkinson's disease (PD) in rats. A total of 24 rats were distributed into 4 equal groups: normal saline control and rotenone control were administered saline or rotenone (ROT), respectively, orally; sterubin 10 received ROT + sterubin 10 mg/kg *po*; and sterubin alone was administered to the test group (10 mg/kg). Rats of the normal saline and sterubin alone groups received sunflower oil injection (*sc*) daily, 1 h after receiving the treatments cited above, while rats of the other groups received rotenone injection (0.5 mg/kg, *sc*). The treatment was continued over the course of 28 days daily. On the 29th day, catalepsy and akinesia were assessed. The rats were then euthanized, and the brain was extracted for estimation of endogenous antioxidants (MDA: malondialdehyde, GSH: reduced glutathione, CAT: catalase, SOD: superoxide dismutase), nitrative (nitrite) stress markers, neuroinflammatory cytokines, and neurotransmitter levels and their metabolites (3,4-dihydroxyphenylacetic acid (DOPAC), dopamine (DA), norepinephrine (NE), serotonin (5-HT), 5-hydroxyindoleacetic acid (5-HIAA), and homovanillic acid (HVA)). Akinesia and catatonia caused by ROT reduced the levels of endogenous antioxidants (GSH, CAT, and SOD), elevated the MDA level, and altered the levels of nitrites, neurotransmitters, and their metabolites. Sterubin restored the neurobehavioral deficits, oxidative stress, and metabolites of altered neurotransmitters caused by ROT. Results demonstrated the anti-Parkinson's activities of sterubin in ROT-treated rats.

## Introduction

Parkinson's disease (PD) is an age-associated neuronal deterioration in the substantia nigra, which secretes dopamine, and is associated with the appearance of Lewy bodies (protein inclusions) in persisting neurons (1,2). The result is a lower level of dopamine (DA) in the striatum, causing a imbalance between DA and acetylcholine levels, which regulate muscle tone and coordination (3). PD leads to motor and non-motor abnormalities in the affected persons (4). Muscle stiffness, bradykinesia, postural instability, and resting tremor are motor indicators of PD (2). Non-motor symptoms include orthostatic hypotension, olfactory impairment, sleep disturbances, and speech impairment (5). In addition, a significant cognitive decline is a common non-motor symptom reported in PD sufferers (6). As the disease advances, other neuronal systems like cholinergic, noradrenergic, and glutanergic are also affected causing further non-motor manifestations such as cognitive decline and depression (7). The mechanism for cell death in PD is not clear, but oxidative stress and neuroinflammation may play a key role (8).

Some factors thought to contribute to PD's etiology and lead to cell death are mitochondrial complex I inhibition, calcium homeostasis, caspase-mediated cell death, impaired metabolism, protein accumulation, and genetic and environmental interactions.

Immune dysregulation in the brain due to the overexpression of neuroinflammatory cytokines triggers a number of pro-inflammatory actions that eventually lead to PD-associated nerve toxicity (9). Tumor necrosis factor (TNF)-α pathways and interferon (IFN)γ receive specific attention in the pathophysiology of PD (5). PD patients are known to have elevated levels of TNF-α in their brain, cerebrospinal fluid, and sera (2). In an experimental study, targeted neutralization of soluble TNF-α signaling dramatically lowered DA cell death, supporting the role of TNF-α in the degeneration of the substantia nigra (5). Moreover, elevated neuroinflammatory cytokines were seen in PD that correlated with severity and impairment level (9). PD is associated with increased free radical damage, which further impairs dopamine metabolism (10).

Despite improvements in the prevention, diagnosis, and treatment of PD, access to suitable management options is restricted by a lack of awareness and public action. There are numerous medications available today used to treat PD, but their use is restrained due to substantial side effects (11). As a result, complementary and alternative medicine is crucial. Natural chemicals are being explored in this area to find a medicine that can prevent nigrostriatal system degeneration (12).

Rotenone (ROT) is a lipophilic compound obtained from the roots of plants belonging to Leguminosae family. It is a natural pesticide, known for its capability to traverse the blood brain barrier. It inhibits mitochondrial complex I of sequential electron transfer with high affinity, leading to oxidative stress and mitochondrial dysfunction in neurons (13). Furthermore, exposure to rotenone causes the selective degeneration of dopaminergic neurons in the substantia nigra, a brain region associated with Parkinson's disease. Rotenone often leads to behavioral changes and motor deficits, such as tremors, rigidity, and bradykinesia (slowness of movement) (14). *Eriodictyon californicum* (Boraginaceae) is traditionally used to treat cold, cough, asthma, bronchitis, headaches, fever, wounds, joint pain, and gastrointestinal problems (15), and has been speculated to possess powerful antioxidant potential (15).

Sterubin is a 7-methoxy-3',4',5-trihydroxyflavanone that has been discovered as the active constituent in *E. californicum*, which belongs to Artemisia family and has powerful anti-inflammatory and neuroprotective actions (15). Sterubin may have anti-oxidant, anti-inflammatory, anti-amyloid, and neurotrophic actions, according to research conducted in cell culture (16). As an anti-oxidant, sterubin can help in neutralizing the harmful free radicals in the body that can cause oxidative stress and damage to cells. The anti-inflammatory effect of sterubin helps in reducing the inflammation in the body that leads to neurodegenerative disorders. Sterubin was found to be a potent neuroprotective compound in a study on mental disorder drugs (16). Sterubin may help enhance the body's immune response, potentially aiding in the defense against infections as a flavonoid. Moreover, sterubin has been shown to have a considerable favorable effect on both retention and abstract memory in mice, protecting the nerve cells and the brain from damage or degeneration (17). Based on these outcomes, it is hypothesized that sterubin helps in PD. The current research aimed to assess the efficacy of sterubin in ROT-induced PD.

## Material and Methods

### Chemicals

Griess reagent, thiobarbituric acid (TBA), and rotenone were obtained from Sigma-Aldrich (USA). Sterubin and pro-inflammatory cytokines (interleukin (IL-6), IL-1β, and TNF-α) kits were obtained from MSW Pharma, India.

### Animals

Wistar male rats, 10-12 weeks old (180±20 g) were maintained under constant parameters following the Institutional Animal Ethics Committee, India (IAEC/TRS/PT/021/005), and the research was conducted as per the ARRIVE guidelines. Rats were maintained in poly-acrylic cages at ambient temperatures, 45-55% humidity, with 12-h light/dark cycles and free access to pellets and water.

### Experimental design

Sterubin diluted in 0.5% sodium carboxymethyl cellulose (SCMC) solution was given orally for 28 days to experimental animals. Sunflower oil was used to emulsify ROT and the mixture was given subcutaneously (0.5 mg/kg) to rats (10).

The normal control received saline with 1 mL of SCMC solution; rotenone control received ROT with 1 mL of SCMC solution; ROT + sterubin 10 received 10 mg/kg *po* sterubin; sterubin alone received sterubin 10 mg/kg *po*.

**Figure 1:**
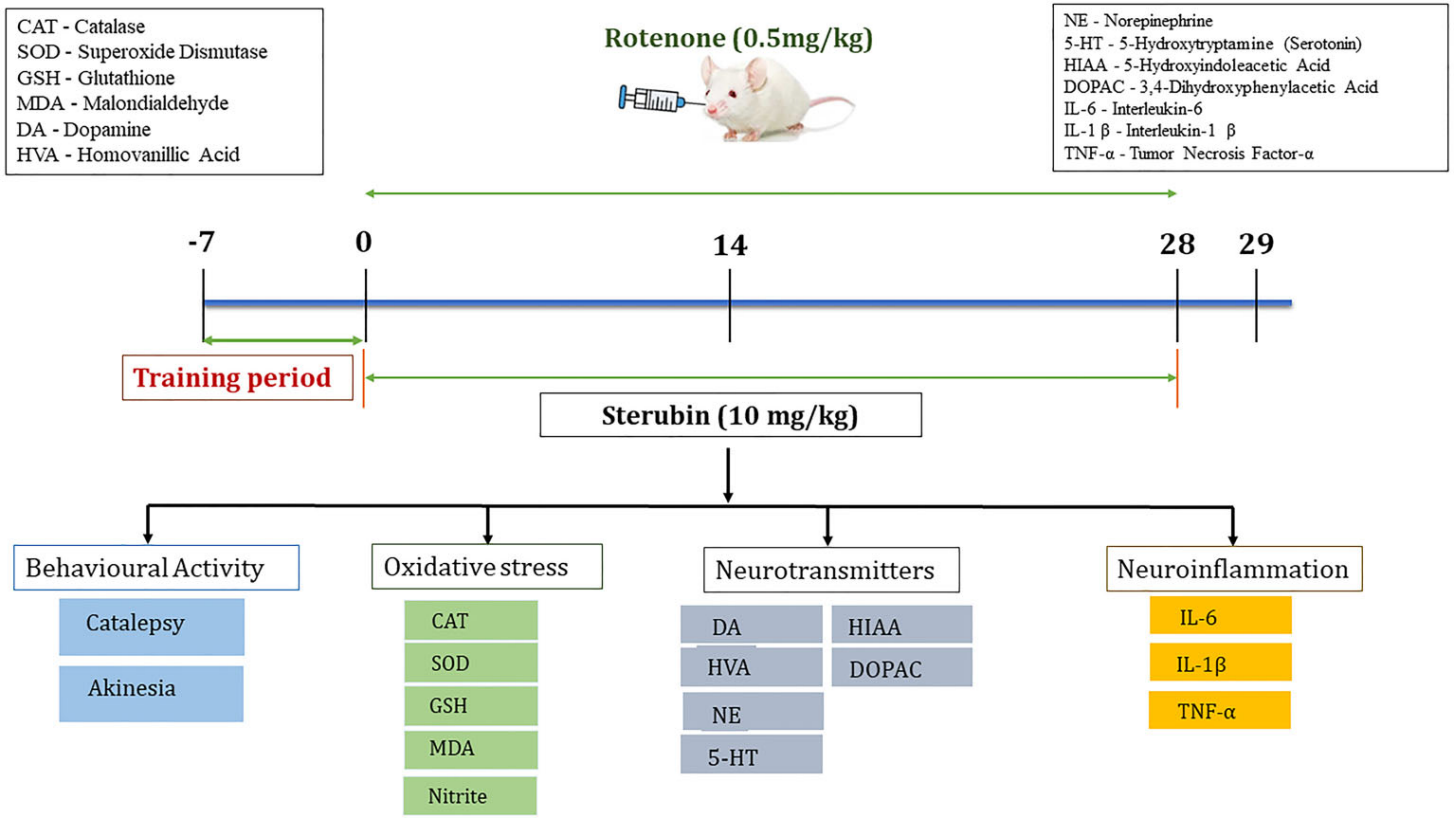
Experimental design.

The proposed treatment plan was continued daily for 28 days. Animals were assessed for catalepsy and akinesia on the 29th day, or 24 h after the final dosage of rotenone. The animals were euthanized, and the brain region was dissected for biochemical analysis. Figure 1 shows the experimental design of the animal model used.

### Behavioral activity

Catalepsy was evaluated using the bar test, which involved the use of a horizontal bar positioned 9 cm above the ground and parallel to it. Rats were positioned with their front paws resting on the bar while in a semi-reared posture. A stopwatch was used to measure the duration it took for the rat to voluntarily remove one of its paws from the bar. A predetermined cutoff time of a maximum of 3 min was set for this assessment (18).

Akinesia was assessed by measuring the time in seconds (s) it took for the animals to mobilize all four of their limbs, with the evaluation completed within a 180-s time limit. Prior to each akinesia test, the rats were allowed to acclimate for 5 min on a wooden platform elevated 100 cm and measuring 100×150 cm (19).

### Biochemical analysis

After immediate dissection, animal brains were isolated and the striatum was separated. Cleaned brain tissue was homogenized in ice-cold phosphate buffer. The supernatant of the tissue suspension was collected and analyzed (20).

#### Reduced glutathione (GSH)

The Ellman method was utilized to measure GSH (21). The brain homogenate was centrifuged at 1000 *g* for 10 min at 4°C after being treated with trichloroacetic acid. Phosphate buffer (3 mL, 0.2 M, pH 8) and 0.5 mL of the DTNB indicator [5-5'-Dithio-bis (2-nitro-benzoic acid)] were added to 1 mL of the isolated supernatant. The spectrophotometric estimate for the reaction mixture was 412 nm. Data are reported in units of nmol GSH per mg protein.

#### Superoxide dismutase (SOD)

SOD activity was measured by observing how much SOD inhibited the spontaneous oxidation of adrenaline to adrenochrome (22). To estimate SOD, NADH was incubated with the brain homogenate for 90 s. Acetic acid and butanol were added to the mixture, and the layer of butanol that was extracted was measured spectrophotometrically at 520 nm (23). Data are reported as nmol/mg protein.

#### Catalase (CAT)

The H_2_O_2_ decomposition method was used (24). In this study, catalase activity at 240 nm was measured by spectrophotometry. A reaction mixture containing 50 mM potassium phosphate buffer (pH 7.0) and 10.5 mM H_2_O_2_ was used. Activation of the enzyme was calculated by measuring the rate at which absorbance at 240 nm decreased after adding the enzyme extract at 25°C for 2 min. A standard graph of H_2_O_2_ was used to determine the units of CAT activity.

#### Malondialdehyde (MDA)

The Wills method was used to determine MDA in the brain of rats. Trichloroacetic acid (10% w/v) and brain homogenate in equal amounts (2 mL) were combined, and after cooling, the flocculent precipitate was removed by centrifugation (3000 *g* for 10 min). Thiobarbituric acid was added to 0.5 mL of supernatant and left in hot water for 15 min. An ultraviolet (UV) spectrophotometer (MSW Pharma, M.S., India) recorded the absorbance at 532 nm and the results are reported as nmol of MDA per mg protein (24,25).

#### Nitrite

The formation of nitric oxide (NO) indicates the accumulation of nitrite in the striatum (22). The Griess method was used for nitrite estimation utilizing a sodium nitrite standard curve, and the results are reported in µg/mL (26).

#### Neurotransmitters and metabolites

The neurotransmitters namely dopamine (DA), homovanillic acid (HVA), norepinephrine (NE), serotonin (5-HT), and its metabolites, 5-hydroxyindoleacetic acid (5-HIAA) and 3,4-dihydroxyphenylacetic acid (DOPAC) were examined by HPLC (12).

#### Neuroinflammatory cytokines

For the determination of the levels of various proinflammatory biomarkers (IL-6, IL-1β, and TNF-α) commercially available ELISA kits (Krishgen Biosystems, M.S., India) were used. An ELISA plate (EIAQuant, Merillife Pvt. Ltd., India) with an antibody coating was filled with the isolated protein. The manufacturer's procedure was followed to quantify the concentration of cytokines, reported in pg/mL (22).

### Statistical analysis

All data are reported as means±SE and were analyzed with Graph Pad Prism 5.0 (USA). One-way analysis of variance (ANOVA) was performed together with Tukey's *post hoc* test for all biochemical and behavioral parameters with limits of P<0.05.

## Results

### Catalepsy

**Figure 2:**
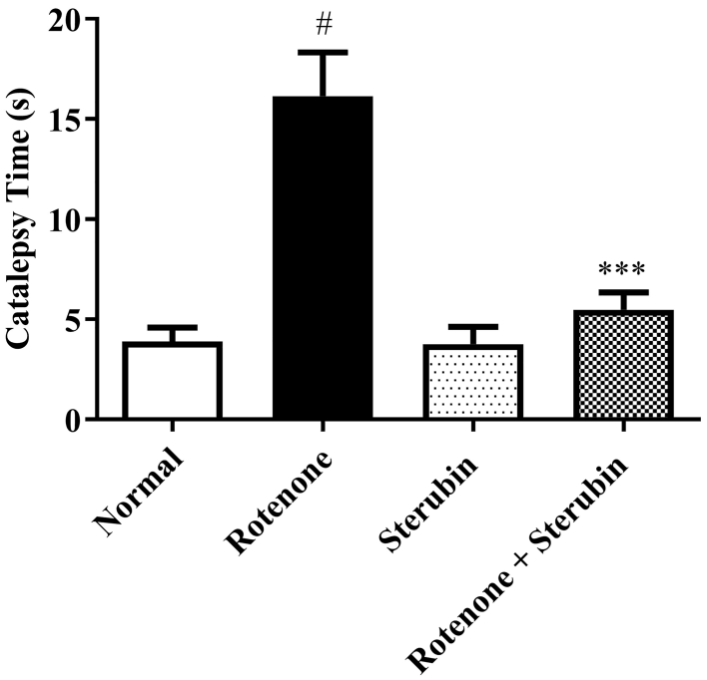
Effect of sterubin (10 mg/kg) on catalepsy in rotenone-treated rats. Data are reported as means±SEM (n=6). ^#^P<0.001 *v*s normal saline control; ***P<0.0001 *vs* rotenone control (ANOVA).

Normal control rats quickly adjusted their posture on the raised bar. The rotenone control rats took significantly longer to remove their paw from the elevated surface compared to the normal control rats (P<0.001). The rotenone with sterubin (10 mg/kg) rats had enhanced muscular strength and the time was significantly reduced [F(3, 20)=16.19, P<0.0001]. No significant differences were found in the sterubin alone group. Figure 2 displays the catalepsy test findings.

### Akinesia

**Figure 3:**
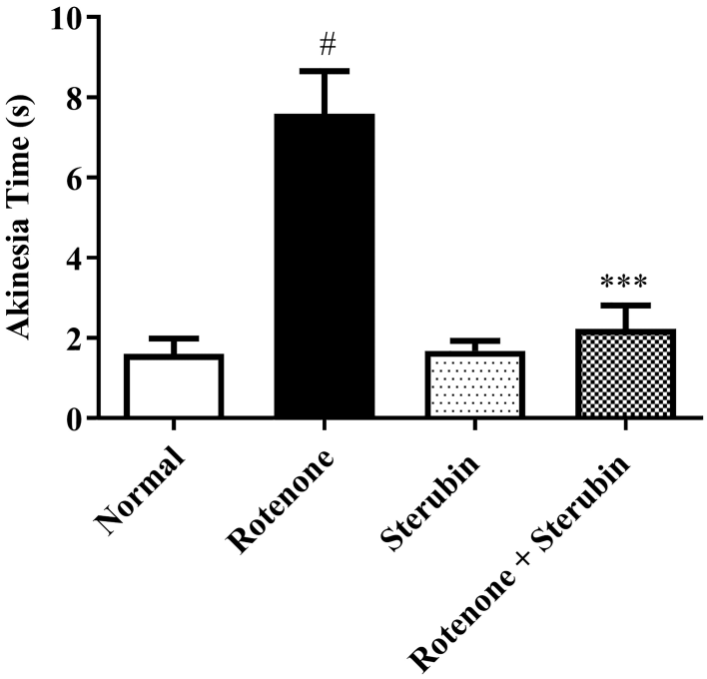
Effect of sterubin (10 mg/kg) on akinesia in rotenone-treated rats. Data are reported as means±SEM (n=6). ^#^P<0.001 *vs* normal saline control; ***P<0.0001 *vs* rotenone control (ANOVA).

Normal control animals mobilized all of their four limbs on the elevated platform normally. Rats treated with rotenone took a longer time (P<0.001) and had difficulty initiating movements. Rotenone-induced difficulties were improved in rats treated with rotenone after sterubin administration [F(3, 20)=15.91, P<0.0001]. Sterubin alone treatment did not affect akinesia in rats. Figure 3 shows the impact of sterubin on akinesia in the groups.

### Endogenous antioxidants

**Figure 4:**
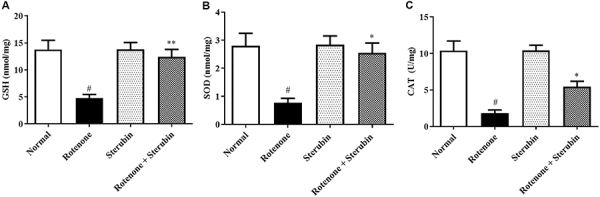
Effect of sterubin (10 mg/kg) on (**A**) reduced glutathione (GSH), (**B**) superoxide dismutase (SOD), and (**C**) catalase (CAT) activities in rotenone-treated rats. Data are reported as means±SEM (n=6). ^#^P<0.001 *vs* normal saline control; *P<0.05 and **P<0.001 *vs* rotenone control (ANOVA).

Endogenous brain antioxidant levels in rats exposed to ROT were depleted. Administration of sterubin (10 mg/kg) to ROT normalized the levels of SOD [F(3, 20)=6.396, P<0.0001], GSH [F(3, 20)=8.735, P<0.0001], and CAT [F(3, 20)=16.45, P<0.0001] compared to ROT control animals. Administration of sterubin alone did not produce any changes compared with the normal saline control (Figure 4A-C).

### MDA and nitrite

**Figure 5:**
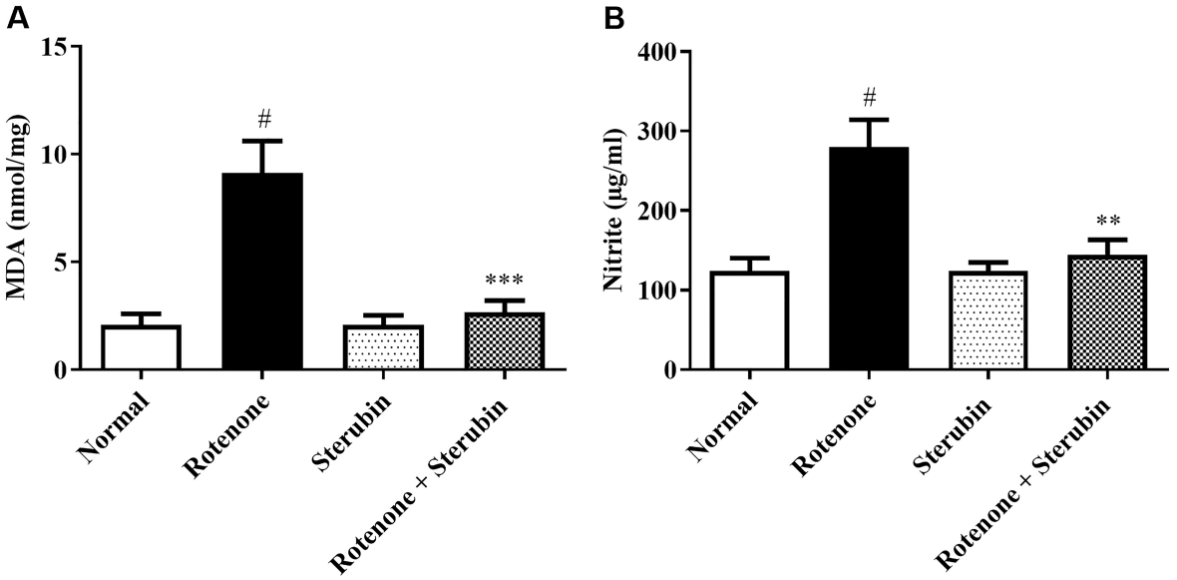
Effect of sterubin (10 mg/kg) on (**A**) malondialdehyde (MDA) and (**B**) nitrite in rotenone-treated rats. Data are reported as means±SEM (n=6). ^#^P<0.001 *vs* normal saline control; **P<0.001 and ***P<0.0001 *vs* rotenone control (ANOVA).

ROT elevated MDA and nitrite levels in rats (P<0.001) and sterubin attenuated the increase in MDA [F (3, 20)=13.22, P<0.0001] and nitrite [F(3, 20)=8.632, P<0.0001] compared to rotenone control rats. Sterubin alone did not induce any changes in MDA or nitrite compared to the normal saline control. The MDA and nitrite results are shown in Figure 5A and B.

### Neurotransmitters and their metabolites

**Figure 6:**
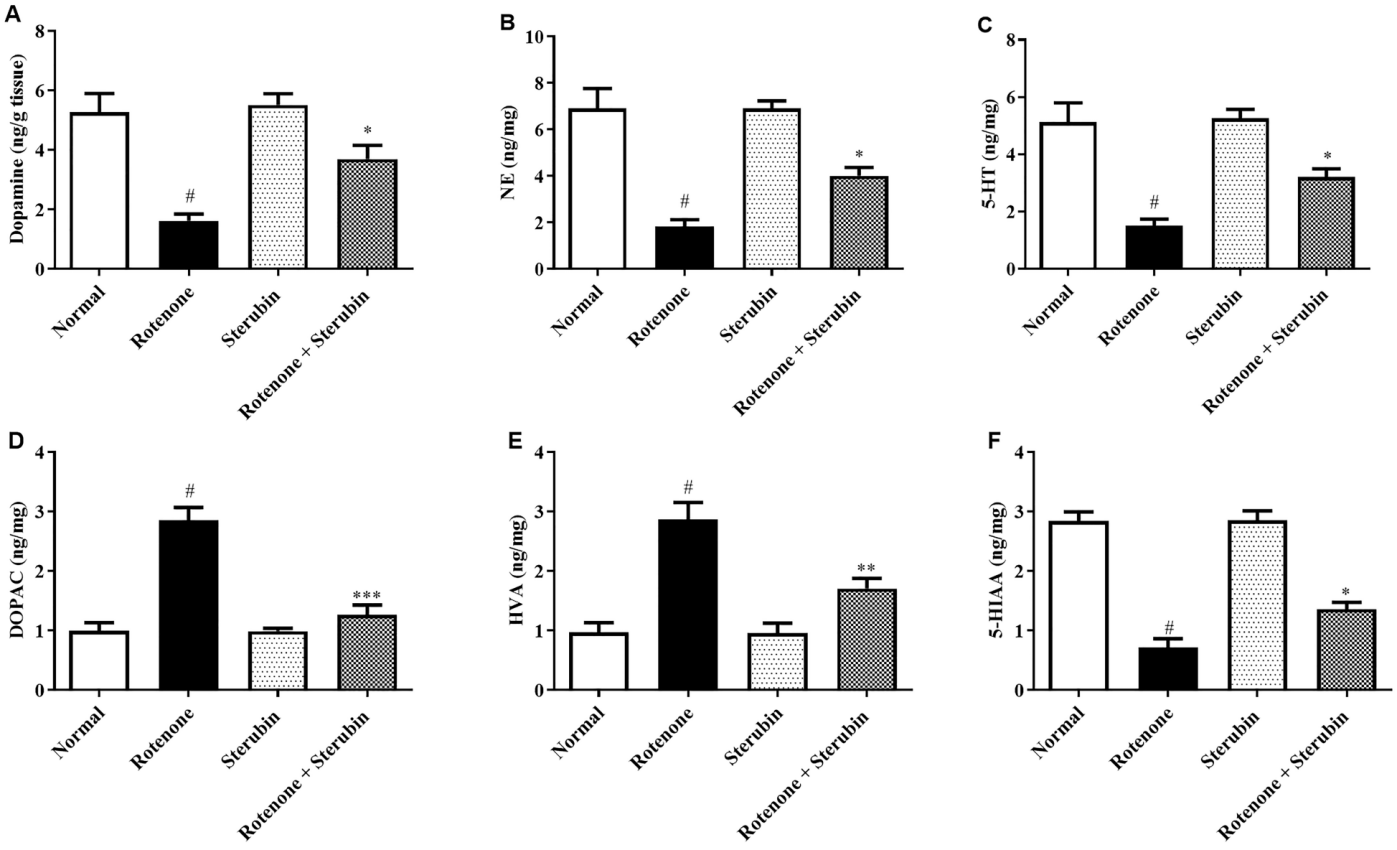
Effect of sterubin (10 mg/kg) on (**A**) dopamine; **B**, norepinephrine (NE); **C**, serotonin (5-HT); **D**, 3,4-dihydroxyphenylacetic acid (DOPAC); **E**, homovanillic acid (HVA); and **F**, 5-hydroxyindoleacetic acid (5-HIAA) in rotenone-treated rats. Data are reported as means±SEM (n=6). ^#^P<0.001 *vs* normal saline control; *P<0.05, **P<0.001, and ***P<0.0001 *vs* rotenone control (ANOVA).

ROT decreased the levels of neurotransmitters such as DA, NE, and 5-HT (P<0.001) in rats compared to the normal saline control. In addition, ROT increased the levels of DOPAC and HVA and decreased 5-HIAA (P<0.001) in the treated animals compared to the normal saline control. Sterubin and ROT injected animals restored DA [F(3, 20)=11.05, P<0.0001], NE [F 3, 20)=16.03, P<0.0001], 5-HT [F(3, 20)=12.70, P<0.0001], HVA [F(3, 20)=14.28, P<0.0001], DOPAC [F(3, 20)=18.16, P<0.0001], and 5-HIAA [F(3, 20)=37.66, P<0.0001] to normal. Sterubin alone did not induce significant alterations in the chemical transmitters and their metabolites. The results of these estimations are shown in Figure 6A-F.

### Neuroinflammatory cytokines

**Figure 7:**
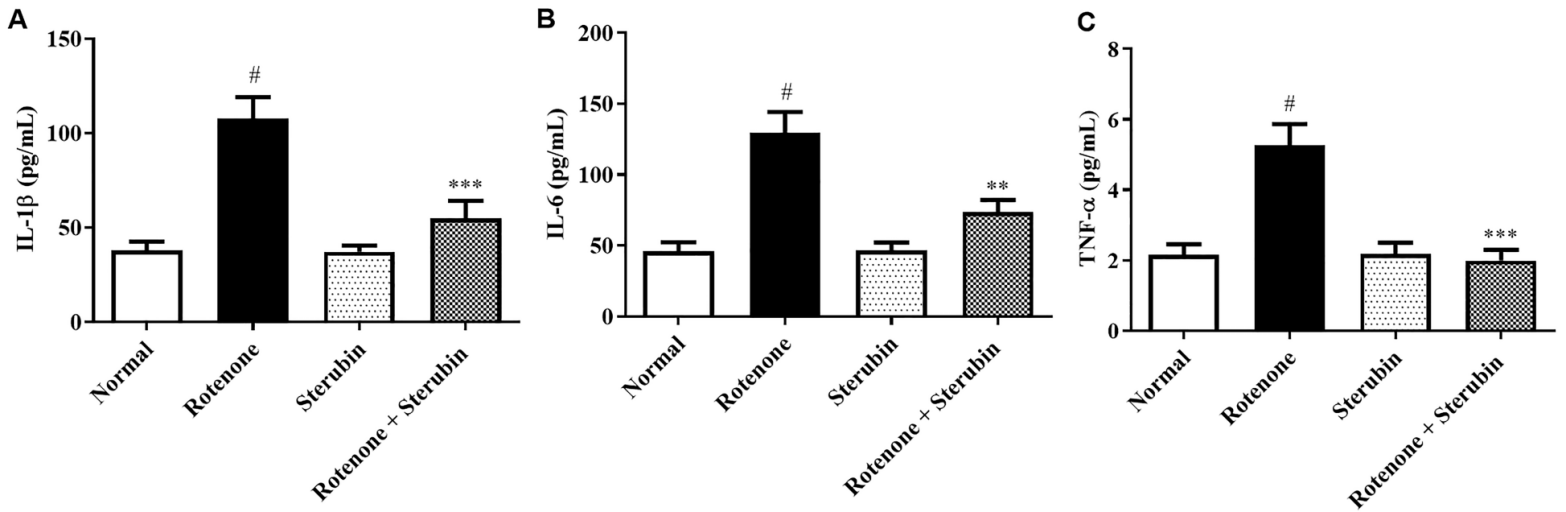
Effect of sterubin (10 mg/kg) on (**A**) interleukin (IL)-1β; **B**, IL-6; and **C**, tumor necrosis factor (TNF)-α in rotenone-treated rats. Data are reported as means±SEM (n=6). ^#^P<0.001 *vs* normal control; **P<0.001 and ***P<0.0001 *vs* rotenone control (ANOVA).

The IL-6, TNF-α, and IL-1β levels were significantly elevated in ROT control animals (P<0.001) compared to normal saline control animals. Administration of sterubin to ROT-injected rats attenuated IL-6 [F(3, 20)=9.576, P<0.0001], TNF-α [F(3, 20)=13.04, P<0.0001], and IL-1β [F(3, 20)=13.23, P<0.0001] towards normal. Sterubin alone did not produce significant changes in neuroinflammatory markers compared to normal saline control rats. The results of neuroinflammatory cytokines are shown in Figure 7A-C.

## Discussion

ROT is a toxin from a plant species that is toxic to humans, animals, and insects (27). It suppresses the enzyme for the mitochondrial respiratory chain complex-I, which hinders NADH oxidation and causes neurotoxicity by selectively exposing dopaminergic neurons to ROT, inducing PD-like symptoms, as dopaminergic neurons are more prone to oxidative assaults (27).

Early signs of PD include muscle rigidity, a shuffling gait, difficulty in movement, and neuronal loss in the part of the brain that controls the locomotor activity (28,29). The outcomes of the present study confirmed the muscular rigidity, muscle control loss, and restricted body motion induced by ROT, as evidenced by severe akinesia and catatonia in rats receiving ROT. Animals treated with ROT who were given sterubin experienced less catatonic behavior and more movement. These findings demonstrated the positive effects of sterubin in ROT-induced muscular rigidity and body movement in animals.

In the animal's brain, ROT increased the level of nitrite and MDA while lowering endogenous antioxidants. Sterubin treatment showed anti-rotenone antioxidant potential by reducing ROT-induced endogenous antioxidant depletion along with oxynitrative stress in the mice. Mitochondrial malfunction and nitroxidative stress are two interconnected pathways that have been identified as key events in the degeneration of neurons in PD (30).

The monoamine oxidase (MAO) enzyme metabolizes dopamine into DOPAC, which is further converted by catechol-O-methyl transferase (COMT) to HVA. The enzyme COMT converts dopamine to 3-MT, which is further converted to HVA by the enzyme MAO (31). Norepinephrine is the third metabolic byproduct of dopamine (32). In the presence of hydrogen peroxide, DOPAC converts to dangerous metabolites that damage vesicles of dopamine storage. This can contribute to the failure of levodopa therapy in PD. MAO-B inhibitors help lessen these complications in people with PD. In this study, administration of ROT led to reduction of DA and NE and elevated DOPAC and HVA levels in the rat brains. Whereas treatment with sterubin in ROT-treated animals improved the intensity of dopamine and norepinephrine and reduced the levels of HVA and DOPAC, indicating that sterubin inhibited MAO-B.

The serotonergic system plays a substantial role in the pathophysiology of PD non-motor symptoms (33,34). As an outcome, 5-HT and its metabolite 5-HIAA may be considered as indicators of the disease (33,34). ROT significantly depleted 5-HT and 5-HIAA in rats, leading to depressive behavior. Sterubin reversed the ROT-induced decrease of 5-HIAA and 5-HT in rats. This indicated the beneficial action of sterubin on ROT-induced non-motor symptoms in rats.

The cause of PD is multifaceted, and inflammation contributes to neurodegeneration. PD patients have higher levels of inflammatory cytokines. The present study results strongly validated the previously stated outcomes in ROT-induced PD. Sterubin attenuated the ROT-induced increase in TNF-α, IL-1β, and IL-6. This confirmed the anti-inflammatory capability of sterubin in ROT-induced neuroinflammation in animals. Further studies are required, including immunohistochemical analysis and western blot, to determine whether sterubin has therapeutic potential in PD.

### Limitations

The small number of animals for the study along with the short duration of recording the outcomes were considered to be a limitation of the study. The study also lacked an experimental group treated with the standard drug (i.e., levodopa) to compare its effects with those of sterubin in mitigating the impact of ROT. This comparison would have provided valuable insights into the relative efficacy of these treatments in the context of Parkinson's disease. Another limitation was that the isolation and examination of specific critical brain regions, such as the prefrontal cortex, hippocampus, and striatum did not allow a deeper understanding of the effect of sterubin. Lastly, the investigation into sterubin's potential role as a MAO-B inhibitor could have provided valuable mechanistic insights into its neuroprotective effects.

### Conclusion

Oxidative stress caused by ROT toxicity appears to be responsible for motor impairments, thus antioxidants may prove beneficial. The current work showed that sterubin reduced ROT-induced akinesia, catalepsy, and metabolic abnormalities in rats by reducing free radical damage and the response towards the inflammatory action. The observed beneficial effects may be due to the inhibitory activities of sterubin on neuroinflammatory cytokines. Sterubin is a potential therapeutic agent against neurodegenerative diseases.
